# Frequent experience with face coverings for 10 months improves emotion perception among individuals with high autistic traits: A repeated cross-sectional study

**DOI:** 10.1177/17470218221135585

**Published:** 2022-12-22

**Authors:** Jia Hoong Ong, Fang Liu

**Affiliations:** School of Psychology and Clinical Language Sciences, University of Reading, Reading, UK

**Keywords:** Emotion perception, autism, perceptual learning, face coverings

## Abstract

Face coverings pose difficulties for emotion recognition, but it is unclear whether improvement in recognising emotions from the eyes is possible with experience and whether this might be dependent on one’s autistic traits, given the associations between high autistic traits and poorer emotion perception and reduced gaze to the eye region. In this preregistered study, participants completed a forced-choice emotion recognition task with photographs of eyes and demographic questionnaires that measure their autistic traits and their interaction frequency with others wearing face coverings at two time points: once at the start of the face covering mandate and again 10 months later. We found that after 10 months, individuals with high autistic traits as a cohort recognised emotions from just the eyes better as a function of their experience with others wearing face coverings, suggesting that emotion perception is malleable even for those who have difficulties with emotion perception.

## Introduction

The widespread use of face coverings during the coronavirus disease 2019 (COVID-19) pandemic has resulted in some anecdotal reports of difficulties with recognising emotions in others who are wearing face coverings. This is not surprising, given that emotion perception is difficult under non-ideal conditions, such as when there is visual noise as demonstrated in previous studies (Kätsyri et al., 2008). More recently, direct evidence of the negative impact of face coverings on emotion perception has been observed for both children (Ruba & Pollak, 2020) and adults (Carbon, 2020; Grundmann et al., 2021): observers tend to have more difficulties recognising emotions from static photographs of expressers with face coverings than those without, presumably because less emotional information can be conveyed through just the eyes than by the whole face. This difficulty was also found among “expert” observers, that is, those who have prior experience with face coverings, such as medical and nursing students (Bani et al., 2021). Although those studies have typically used stimuli that have been digitally manipulated (i.e., by adding face coverings to the images), and so that, one might argue that the findings may be an artefact of such manipulation, a recent study has shown that this is unlikely the case as the authors replicated the findings using real photographs of the same expressers with and without face coverings (Fitousi et al., 2021). Thus, the negative impact of face coverings on emotion perception appears to be robust.

Autism spectrum condition (ASC) is a neurodevelopmental condition that is characterised by difficulties with social communication and interaction as well as restricted and repetitive patterns of behaviours or interests (American Psychiatric Association, 2013). Although not a formal diagnostic criterion, autistic individuals^1^ generally have more difficulties with emotion perception than neurotypical individuals (Leung et al., 2022; Uljarevic & Hamilton, 2013), and some propose that this difficulty exacerbates autistic individuals’ social interaction challenges (Williams & Gray, 2013). We suspect that autistic individuals may have even more difficulties recognising emotions of expressers wearing face coverings based on the “eye-avoidance hypothesis” (Tanaka & Sung, 2016), according to which autistic individuals tend to avoid looking at one’s eye region because eyes are perceived to be socially threatening. Supporting the hypothesis, a review on emotion perception studies that measured eye-tracking found that autistic individuals do indeed show reduced gaze to the eyes of emotional faces (Black et al., 2017). Thus, if the eye-avoidance hypothesis were true, then, relative to neurotypical individuals, autistic individuals would be even more constrained by the limited emotional information conveyed by someone wearing a face covering. This was examined directly in a recent study, though, contrary to the eye-avoidance hypothesis, the authors found no effect of autistic traits on emotion recognition accuracy (Pazhoohi et al., 2021). However, there are several methodological concerns in that study that need to be addressed. First, participants’ autistic traits were measured using the 10-item autism-spectrum quotient (AQ-10) (Allison et al., 2012), which is arguably less sensitive than the full 50-item version (Baron-Cohen, Wheelwright, Skinner, et al., 2001). The psychometric properties of AQ-10 have been questioned, specifically its internal reliability and validity (Jia et al., 2019; Taylor et al., 2020). Moreover, the participants were drawn from the university student and general population, and it is unclear how many of them have an ASC diagnosis (this was not reported in the study). Thus, it remains to be seen if autistic individuals or individuals with high levels of autistic traits have more difficulties recognising emotions of someone wearing a face covering than neurotypical individuals or individuals with lower levels of autistic traits.

Despite the difficulties with emotion perception under non-ideal situations or due to individual characteristics, emotion perception is malleable and can be trained. Among neurotypical individuals, this has been demonstrated using lab-based training on challenging stimuli (e.g., stimuli with subtle expressions, with visual noise) (Du et al., 2016; Plate et al., 2019) and through long-term passive exposure, such as childhood maltreatment and bullying, which results in recognition biases for certain emotions (e.g., children who have been abused recognise fear and anger more accurately than those who have not) (Franzen et al., 2021; Pollak & Kistler, 2002). Among autistic individuals, lab-based interventions appear to improve their emotion perception at least immediately after the intervention (Davidson et al., 2022; Zhang et al., 2021), but it is unclear if such changes are possible through long-term passive exposure (i.e., without proper explicit feedback).

In this preregistered study, we took advantage of the widespread adoption of face coverings during COVID-19 to examine this research question. Specifically, we investigated whether we can perceptually learn to recognise emotions from just the eyes as we gain more experience with others wearing face coverings, and whether the improvement (if any) is dependent on one’s level of autistic traits. To that end, participants in the United Kingdom completed an emotion recognition task with photographs of pairs of eyes as stimuli at two time points spaced 10 months^2^ apart: Wave 1 in September 2020 (i.e., approximately 1 month after face coverings became mandatory in the United Kingdom), and Wave 2 in July 2021. We predicted that across both waves, participants with lower levels of autistic traits would show greater improvement in their ability to recognise emotions from the eyes than participants with higher levels of autistic traits.

The data and the code for this study are publicly accessible at https://osf.io/xn3g4/. The materials are not publicly accessible. There is a preregistration for this study at https://osf.io/5kvnp. Deviations from the preregistration are noted in this article.

## Method

### Participants

A total of 308 adults (*M*_age_ = 21.98, *SD* = 9.36, range = 16–66; 234 females, 68 males, and six other/non-binary) participated in the study in Wave 1 (September 2020). Their scores on the 50-item AQ, which measures their autistic traits, ranged between 0 and 49 (*M* = 20.24, *SD* = 8.70). About 10% of the participants (*n* = 29) self-reported to have ASC. In Wave 2 (July 2021), 258 adults (*M*_age_ = 25.44, *SD* = 10.14, range = 16–66; 208 females, 42 males, and eight other/non-binary) participated in the study. Similar to Wave 1, the AQ scores in Wave 2 ranged between 0 and 46 (*M* = 21.21, *SD* = 9.61) and approximately 10% of the participants (*n* = 27) self-reported to have ASC. We identified a subset of participants (*n* = 32) who completed both waves. Additional participants (Wave 1, *n* = 16; Wave 2, *n* = 52) were tested but were excluded from data analysis because they did not pass the attention checks, that is, scoring below 65% on the catch trials.^3^

Participants were recruited from our own participant database, psychology research participant pool, social media, and word-of-mouth. All participants provided their informed consent prior to participating, and they received course credit or entered a lucky draw for vouchers as reimbursement. The study protocol was reviewed and approved by the University Research Ethics Committee (UREC) at the University of Reading.

### Materials and tasks

Participants in Waves 1 and 2 completed the following tasks: a demographic questionnaire, the AQ, and an emotion recognition task. Participants in Wave 2 additionally completed the Toronto Alexithymia Scale (TAS), which was not preregistered as it was added after Wave 1 data collection.

#### Demographic questionnaire

We collected basic demographic information, such as age, gender, and whether they have a diagnosis of ASC. Crucially, participants were asked how often they interacted with others wearing face coverings on a 5-point scale (*never*, *rarely*, *sometimes*, *often*, and *always*). We used participants’ responses to this question to examine whether interaction with others wearing face coverings will lead to improvement in emotion recognition, consistent with a recent study demonstrating that adults with the most social interaction before and after the mask mandate (and thus, most experience with face coverings) showed the largest increase in the use of facial cues in emotion recognition (Barrick et al., 2021).

#### Autism-spectrum quotient

Participants responded to all 50 items of the AQ (Baron-Cohen, Wheelwright, Skinner, et al., 2001), which assess their autistic traits, by indicating how much they agree each item applies to them (e.g., “I prefer to do things the same way over and over again.”) on a 4-point scale (*definitely agree*, *slightly agree*, *slightly disagree*, and *definitely disagree*). Higher AQ scores indicate higher levels of autistic traits.

#### Emotion recognition task

In this forced-choice emotion recognition task, participants were shown a grey-scale photo of a pair of eyes on every trial, and they had to choose the label that best expresses the emotion in the photo without any time limit. We used two sets of emotions: basic and complex. Images for the basic emotion condition (angry, disgusted, happy, fearful, sad, and surprised) were taken from the EU-Emotion Stimuli data set (O’Reilly et al., 2016). For these trials, participants were given six labels to choose from corresponding to the six basic emotions examined in the study. Images for the complex emotions were taken from the Reading the Mind in the Eyes task (RMET) (Baron-Cohen, Wheelwright, Hill, et al., 2001), which measures various mental states (e.g., interested, hostile, playful). Although conceived as a measure of theory of mind, some have argued that the RMET is better thought of as a task that measures emotion recognition ability (Oakley et al., 2016). Following the RMET task protocol, and different from the basic emotion trials, participants chose the most appropriate label from four options.

Trials from both sets of emotions were intermixed and randomly ordered, and all participants completed the same order.^4^ In all trials, participants were given a glossary that provided definitions for all the emotion labels. As attentional check, photos with emotional labels printed on them were presented and participants were explicitly asked to choose that label.

#### Toronto Alexithymia Scale

Wave 2 participants completed the TAS (Bagby et al., 1994), a measure of alexithymia, or a disorder characterised by difficulties expressing and identifying emotions. Participants indicated how much they agree to 20 items (e.g., “I am often confused about what emotion I am feeling.”) on a 5-point scale (*strongly disagree*, *moderately disagree*, *neither disagree nor agree*, *moderately agree*, and *strongly agree*). Higher TAS scores indicate higher alexithymic traits.

### Procedure

Participants completed the study in the following order: demographic questionnaire, AQ, TAS (for Wave 2 participants), and emotion recognition task. The entire study took approximately 30 min to complete. Data collection was conducted for 3 months during each wave (Wave 1: September–December 2020; Wave 2: July–October 2021).

### Data analysis

We fitted a binomial mixed effects model using the *lme4* package (Bates et al., 2015), given the binary dependent variable (correct/incorrect), with the following fixed effects: wave (1 vs 2); emotion (basic vs complex); experience with others wearing face coverings (hereafter, “Face Covering”; rarely vs sometimes vs often); autistic traits (AQ), and all the possible interactions. The face covering variable was recoded to three levels instead of the five stated in the preregistration due to an uneven distribution of participants across the five levels (i.e., those who responded “never” or “rarely” had their responses recoded as “rarely”; and those who responded “often” or “always” were recoded as “often.” The “sometimes” responses were not recoded). Due to the widespread mask mandate between the two time points, we assumed that those in Wave 2 would generally have had more experience with face coverings than those in Wave 1, and so it follows that each face covering level in Wave 2 would not be numerically the same as its corresponding level in Wave 1. We believe that these assumptions are fair given that the majority of the UK residents (95%) reported wearing face coverings when outside even after the relaxation of the mask mandate in July 2021 (i.e., during Wave 2 data collection) (Office for National Statistics, 2021). From this, we can infer that compliance to the mask mandate was high, and so it is safe to assume that participants would have had more encounters with face coverings in Wave 2 than Wave 1 generally. All the categorical predictors were effect-coded, and the continuous variable (AQ) was mean centred by wave. As random effects, we entered random intercept for participant and item, and random by-participant slope for emotion. *p*-values of each predictor was determined using the *Anova()* function from the *car* package (Fox & Weisberg, 2019)—this differed from the preregistration of using the *afex* package (Singmann et al., 2019) because we found that the *afex* package was computationally slower and was more prone to convergence issues. Subsequent post hoc comparisons were conducted using the *emmeans* package (Lenth, 2019). We ran the same binomial mixed effects model twice: once on all participants, and again on a subset of those who did both waves.

Following our preregistration, to confirm our mixed model findings, we analysed the data using an analysis of variance (ANOVA), the output of which can be found in Supplementary Section S1. Findings of the ANOVA were identical to that of the mixed effects model reported in the “Results” section. We also analysed the data from Wave 1 only as a sanity check to replicate expected findings (e.g., the negative relationship between autistic traits and emotion recognition), which can be found in Supplementary Section S2.

## Results

### All participants

The full output is displayed in Table 1. There was a significant Emotion × Wave interaction, χ^2^(1) = 7.29, *p* = .007, and follow-up comparisons revealed that participants’ recognition accuracy did not differ between waves for basic emotions (*z* = 0.90, *p* = .366), but they improved from Waves 1 to 2 for complex emotions (*z* = 2.22, *p* = .027).

**Table 1.** Mixed effects model results on the entire sample.

	χ^2^	*df*	*p*
Intercept	61.09	1	<.001
Emotion	0.86	1	.355
Face covering	1.64	2	.441
AQ	14.42	1	<.001
Wave	0.65	1	.421
Emotion × Face Covering	1.95	2	.377
Emotion × AQ	1.22	1	.269
Face Covering × AQ	0.53	2	.766
Emotion × Wave	7.29	1	.007
Face Covering × Wave	1.64	2	.441
AQ × Wave	0.42	1	.519
Emotion × Face Covering × AQ	0.28	2	.869
Emotion × Face Covering × Wave	1.02	2	.601
Emotion × AQ × Wave	2.49	1	.115
Face Covering × AQ × Wave	7.69	2	.021
Emotion × Face Covering × AQ × Wave	0.84	2	.656

AQ: autism-spectrum quotient.

**Figure 1.**
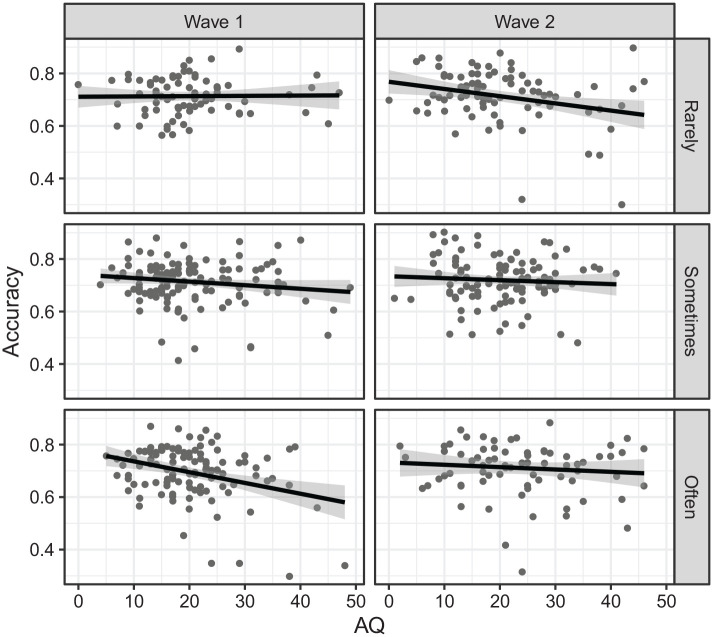
Accuracy on the emotion recognition task as a function of autistic traits (AQ) by wave (Wave 1 vs Wave 2) and experience with others wearing face coverings (rarely vs sometimes vs often) for the entire sample.

There was also a significant negative effect of AQ, χ^2^(1) = 14.42, *p* < .001; *B* = –0.01, *SE* = 0.00, and importantly, a significant interaction involving face covering, AQ, and wave, χ^2^(2) = 7.69, *p* = .021 (see Figure 1). Examining the effect of AQ at each level of face covering by wave revealed a significant negative AQ effect for rarely Wave 2 (*z* = 2.77, *p* = .006), sometimes Wave 1 (*z* = 2.03, *p* = .042), and often Wave 1 (*z* = 3.60, *p* < .001). Pairwise comparisons between waves at each level of face coverings revealed significant difference in the effect of AQ (i.e., difference in slope) only for the often condition, with the AQ effect less negative in Wave 2 than in Wave 1 (*z* = 2.28, *p* = .023).

### Subset of participants who participated in both waves

The same binomial mixed effects model was fitted to data from a subset of participants who completed both waves (see Table 2 for the full output), although note that given the small sample size (*n* = 32), the results should be interpreted with caution.

**Table 2.** Mixed effects model results on a subset of participants who completed both waves (*n* = 32).

	χ^2^	*df*	*p*
Intercept	43.40	1	<.001
Emotion	1.35	1	.245
Face covering	1.50	2	.472
AQ	4.89	1	.027
Wave	0.34	1	.560
Emotion × Face Covering	0.27	2	.875
Emotion × AQ	5.54	1	.019
Face Covering × AQ	1.18	2	.555
Emotion × wave	0.71	1	.401
Face Covering × Wave	0.39	2	.823
AQ × Wave	0.79	1	.374
Emotion × Face Covering × AQ	3.10	2	.212
Emotion × Face Covering × Wave	4.90	2	.086
Emotion × AQ × Wave	0.20	1	.653
Face Covering × AQ × Wave	2.35	2	.309
Emotion × Face Covering × AQ × Wave	3.61	2	.165

AQ: autism-spectrum quotient.

**Figure 2.**
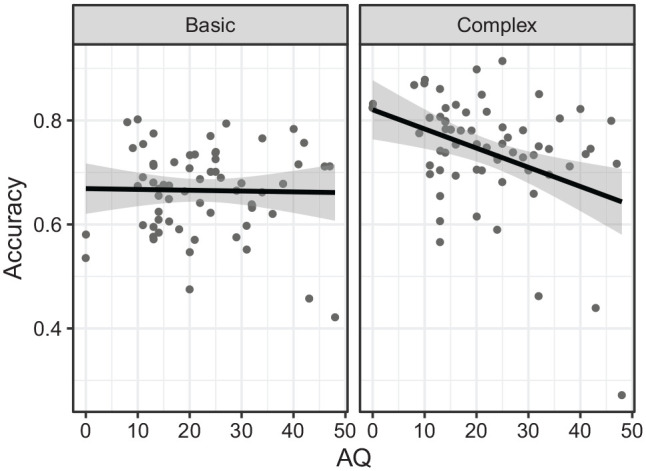
Accuracy on the emotion recognition task as a function of autistic traits (AQ) by emotion (basic vs complex) for a subset of participants who completed both waves.

Similar to the analysis with the entire sample, there was a significant negative effect of AQ, χ^2^(1) = 4.89, *p* = .027; *B* = –0.01, *SE* = 0.01. There was also a significant interaction between emotion and AQ, χ^2^(1) = 5.54, *p* = .019, with the negative effect of AQ only significant in the complex emotions (*z* = 3.16, *p* = .002) and not in the basic emotions (*z* = 0.74, *p* = .451), and the difference in AQ effect between the emotions was significant (*z* = 2.35, *p* = .019, see Figure 2).

**Figure 3.**
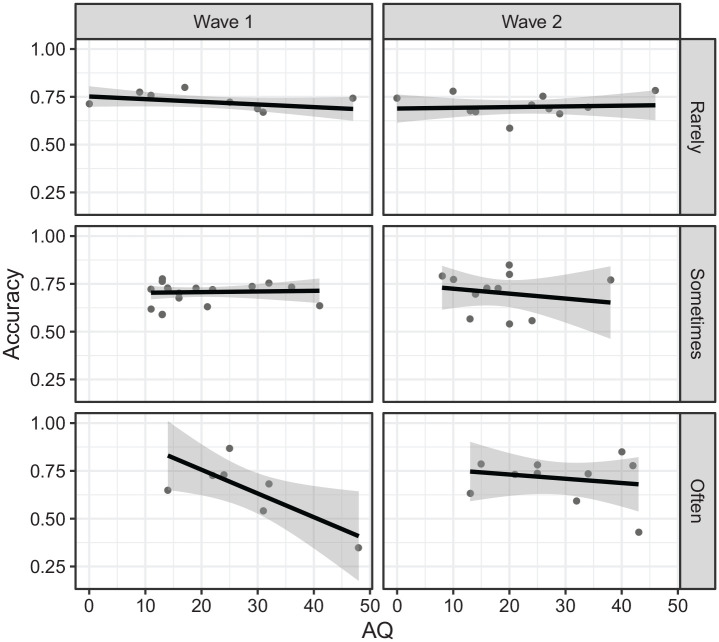
Accuracy on the emotion recognition task as a function of autistic traits (AQ) by wave (Wave 1 vs Wave 2) and experience with others wearing face coverings (rarely vs sometimes vs often) for a subset of participants who completed both waves.

Unlike the analysis with the entire sample, the crucial three-way interaction of Face Covering × AQ × Wave was not significant, χ^2^(2) = 2.35, *p* = .309. We explored the interaction nonetheless and found that the effect of AQ at each level of face covering and wave was only significant for often Wave 1 (*z* = 2.34, *p* = .019, see Figure 3), but none of the pairwise comparisons of the AQ effect between waves at each level of face covering were significant.

## Discussion

The widespread adoption of face coverings, although effective in combatting airborne infections (Worby & Chang, 2020), negatively affects one’s ability to recognise emotions in others wearing face coverings (Bani et al., 2021; Carbon, 2020; Fitousi et al., 2021; Grundmann et al., 2021; Ruba & Pollak, 2020), presumably because face coverings limit the emotional information one can perceive (i.e., just through the eyes). It is unclear, however, whether our ability to recognise emotions from just the eyes can be improved as we gain more experience with face coverings and whether this improvement may be dependent on one’s autistic traits, given that autistic individuals or individuals with high levels of autistic traits tend to have poorer emotion recognition ability generally and avoid looking at the eye region (Tanaka & Sung, 2016).

We examined those questions directly in the present study by examining adult participants’ ability to recognise emotions from photographs of eyes at the start of face covering mandate and again 10 months later. We found that emotion recognition ability was generally poorer among those with high autistic traits, consistent with previous studies (Leung et al., 2022; Uljarevic & Hamilton, 2013). We additionally found that individuals with high autistic traits had better emotion recognition ability from the eyes as a function of their experience with others wearing face coverings after 10 months such that the more interactions they had with others wearing face coverings, the better their recognition performance. This implies that despite initial difficulties with recognising emotions through eyes, given sufficient exposure and experience, autistic individuals may improve in their ability to do so, consistent with the idea that emotion perception is malleable even for individuals who have difficulties with emotion perception.

Analysis on Wave 1 data alone (see Supplementary Section S2) showed that, after about 1 month of the mask mandate, the negative effect of AQ was only observed among those who often encountered others wearing face coverings, whereas, no such AQ effect was found among those who rarely encountered others wearing face coverings. At first glance, this appears contradictory to our general conclusion. However, we caution against drawing too strong a conclusion across the different face covering groups based only on findings from Wave 1. This is because: (1) it is unlikely that 1 month of passive exposure alone is sufficient to drive any significant changes to one’s emotion recognition ability, particularly because the exposure to face coverings would likely be brief and without any explicit feedback on whether the inferred emotion is correct and (2) participants in the different face covering groups may reflect different sub-populations. For example, high autistic traits individuals who often encounter face coverings may have certain inherent qualities that would benefit from being around others wearing face coverings relative to high autistic traits individuals who rarely encounter face coverings. These inherent qualities may include, for instance, having (more) underlying co-morbidity or health conditions, such as anxiety disorders (Croen et al., 2015), which may exacerbate their emotion perception difficulties (Kret & Ploeger, 2015). Thus, we believe that comparing the AQ slope in Wave 1 across the different face coverings groups might not be appropriate as it would be comparing different sub-populations.

The crucial finding was only observed in the main analysis that consisted of mostly different participants across both data collection waves and not in the subset analysis of the small group of participants who completed both waves. Concerning the former, our study design is best characterised as a repeated cross-sectional design, given that most participants across both waves were different individuals. Such a study design is not uncommon in psychology, and some reported similar findings when directly compared findings from a repeated cross-sectional design with that of a panel/longitudinal design (Butterworth et al., 2020; Caplan et al., 1995; Sommet et al., 2018; Zettler et al., 2021). Although it is difficult to establish within-subject improvement in repeated cross-sectional studies, we argue that because we recruited participants in both waves in the same manner, and thus, sampled from the same population, we can make inferences about changes in that population as a cohort. Note also that the use of random intercepts for participants in our analyses means that we considered any variance in the average “starting level” of emotion recognition performance between participants. The subset data showed a similar trend to the main analysis in that, among those who often have experience with others wearing face coverings, the effect of AQ was significantly negative in Wave 1 but not in Wave 2. However, the crucial interaction was not significant, which, we speculate, is likely due to the lack of statistical power. Indeed, given that we only managed to link data across waves for 32 participants,^5^ after being stratified to one of the three levels of face covering experience, the effect of AQ was estimated based on approximately 10 participants per level (see Figure 3), and so that, any conclusion drawn must be interpreted with caution.

It should be noted that the improvement was only observed for individuals with high autistic traits, which raises the question of why this was not observed among those with low autistic traits as hypothesised. We speculate that this may be due to limitations of using static photographs as stimuli; despite experience with face coverings, individuals with low autistic traits may not show any further improvement in their ability to recognise emotions from photographs of eyes because the limited emotional information from static photographs of eyes may have constrained their performance (i.e., the stimuli may not be sensitive enough to detect any changes in emotion recognition ability among those with low autistic traits). In real life situations, one may rely on other emotional cues, such as the crinkling of the nose, movement of the face covering, and verbal cues to facilitate emotion recognition in others wearing face coverings, all of which are not present in the stimuli in this study. Indeed, when dynamic emotional cues are present, participants can recognise emotions of expressers with and without face coverings equally well (Kastendieck et al., 2022), suggesting the importance of these cues when interacting with someone with a face covering. Note, however, that the study only examined happy and sad expressions, which may be relatively easy to recognise from just the eyes without those dynamic cues. Thus, the generalisability of the importance of dynamic cues to other emotions as a function of experience with face coverings should be examined in future studies. The static stimuli may be less of an issue for those with high autistic traits because they had more room for improvement in their emotion recognition ability (and indeed, analysis on Wave 1 data alone, reported in Supplementary Section S2, showed poorer performance among those with high autistic traits relative to those with low autistic traits). That is, we speculate that the use of static stimuli is sensitive enough to detect coarse improvement by those with high autistic traits, but not the subtle improvement by those with low autistic traits who already had relatively high recognition performance, across waves.

Putting the limitations of the static stimuli aside, our findings appear contradictory to the eye-avoidance hypothesis (Tanaka & Sung, 2016). According to the hypothesis, autistic individuals or individuals with high autistic traits generally avoid the eye region of the person with whom they are interacting as the eyes are perceived to be socially threatening. If true, then there should be no improvement in their recognition ability across waves, which was not what we found. In light of our findings, we interpret that while it is possible that individuals with high autistic traits may generally avoid the eye region, in the absence of other possible facial cues (e.g., because of face coverings), they do look at the eye region as they gain more experience being in situations that necessitate that. We thus speculate that this eye-avoidance strategy is more of a bias, rather than an absolute, among individuals with high autistic traits. There may still be qualitative differences in how individuals with high autistic traits look at the eye region of one wearing a face covering relative to those with low autistic traits, which can only be determined in future studies using eye-tracking paradigms (e.g., by comparing the number and duration of fixations while looking at images of someone wearing a face covering).

In this study, we examined the influence of autistic traits rather than autism diagnosis on emotion recognition ability. Although the former is widely used, especially in online studies, the two should not be conflated as cautioned by autism researchers (Sasson & Bottema-Beutel, 2022), given that autism diagnosis is typically determined by clinicians from interviews and examining the individual’s history, which is arguably more comprehensive than a self-report questionnaire. We thus repeated the analysis with self-reported autism diagnosis (i.e., comparing those who reported having a clinical diagnosis of autism vs those who did not) and we found similar findings (see Supplementary Section S3): similar to the Face Covering × AQ × Wave seen in the main analysis, there was a significant interaction involving Face Covering × Diagnosis × Wave, which was driven by an improvement from Waves 1 to 2 only among autistic participants who often had interactions with others wearing face coverings. We acknowledge, however, that due to the data collection method, we were unable to confirm their autism diagnosis, but the convergence of findings for both approaches is reassuring.

Some argue that alexithymia, a disorder characterised by difficulties expressing and identifying emotions, rather than autism is the cause for emotion perception difficulties among autistic individuals (Bird & Cook, 2013; Ola & Gullon-Scott, 2020), given the high prevalence of alexithymia among autistic individuals (Kinnaird et al., 2019). Others, however, suggest that *both* autism and alexithymia contribute significant unique variance in emotion perception (Keating et al., 2022; Stephenson et al., 2019). Although the present study is limited by our measurement of alexithymia in Wave 2 only (which was added after Wave 1 data collection and therefore not preregistered), we analysed the data from Wave 2 to determine whether autistic traits or alexithymia affected emotion perception in our study (see Supplementary Section S4). We found both autistic traits and alexithymia separately interacted with emotion and face covering, suggesting that even when alexithymia is accounted for, autistic traits do contribute unique variance in emotion perception. However, it is unclear whether autistic traits and/or alexithymia contribute to emotion perception *improvement* following face covering experience among those with high autistic traits, which should be followed up in future studies.

In conclusion, after 10 months, individuals with high autistic traits had better emotion recognition performance from just the eyes as a function of their experience with others wearing face coverings. We are unable to establish within-individual improvement due to the study design (i.e., repeated cross-sectional with few overlapping participants across both data collection waves) and the subset analysis of those who did both waves lacked statistical power. Nonetheless, because of identical sampling procedure for both waves (i.e., we sampled the same population), we conclude that there is such a general improvement as a cohort. Consistent with previous findings on the malleability of emotion perception, this suggests that long-term passive exposure can modify our emotion perception ability, even among those who have difficulties with perceiving emotions in others.

## Supplemental Material

sj-pdf-1-qjp-10.1177_17470218221135585 – Supplemental material for Frequent experience with face coverings for 10 months improves emotion perception among individuals with high autistic traits: A repeated cross-sectional study Click here for additional data file. Supplemental material, sj-pdf-1-qjp-10.1177_17470218221135585 for Frequent experience with face coverings for 10 months improves emotion perception among individuals with high autistic traits: A repeated cross-sectional study by Jia Hoong Ong and Fang Liu in Quarterly Journal of Experimental Psychology

## Notes

1 We respectfully use the term “autistic individuals,” a term that is preferred by most autistic individuals and their family, to refer to individuals with ASC (Kenny et al., 2016).

2 Wave 2 data collection was originally planned to be 12 months after Wave 1, as stated in the preregistration, but due to changes in the face covering rules in the United Kingdom in July 2021, we moved the data collection period earlier.

3 We increased the attentional check accuracy threshold from 50% as stated in the preregistration to 65% as we found that some of the responses appear to be non-human bots, and therefore, we decided to take a more conservative approach to exclude responses.

4 Trial orders were not completely randomised across participants due to limitations from the platform used to host the study. However, similar to standardised questionnaires with different subscales that have the same randomised item order for all participants, we do not anticipate any major order effects that would affect the general conclusion of the study.

5 It is unfortunate that we could not link data across waves from more participants, and we speculate that this may be due to the following reasons: (1) participants were given the opportunity to “opt-in” to be contacted for Wave 2 and not all the participants did; (2) Wave 2 data collection happened during summer break, during which the participants from the Psychology research participant pool were unlikely to be active; and (3) we requested participants to generate a unique code identifier following our instructions—which we explained would be used to link data across waves—but not all participants did.
