# Supplementary material for: Frequent experience with face coverings for 10 months improves emotion perception among individuals with high autistic traits: A repeated cross-sectional study
Source: Q J Exp Psychol (Hove). 2022 Dec 22;76(7):1599–608. doi: 10.1177/17470218221135585 (PMC10280663; doi:10.1177/17470218221135585)
Supplement: sj-pdf-1-qjp-10.1177_17470218221135585 – Supplemental material for Frequent experience with face coverings for 10 months improves emotion perception among individuals with high autistic traits: A repeated cross-sectional study [file sj-pdf-1-qjp-10.1177_17470218221135585.pdf]

Frequent experience with face coverings for 10 months improves emotion perception among individuals with high autistic traits: A repeated cross-sectional study

Jia Hoong Ong[1] and Fang Liu[1]

[1]School of Psychology and Clinical Language Sciences, University of Reading, Reading, United Kingdom

[SUPPLEMENTARY SECTIONS]

Section S1: Data Analysis using ANOVA

Section S2: Analysis on Wave 1 Data

Section S3: Data Analysis using Autism Diagnosis

Section S4: Analysis on Wave 2 Data using Autistic Traits and Alexithymia Scores

Section S1: Data Analysis using ANOVA

Following the study preregistration protocol, we analysed the data using an ANOVA, given that it is commonly used in emotion perception research though we recognise the limitations of doing so given the binary nature of the dependent variable (Jaeger, 2008). Similar to the mixed effects model, we entered autistic traits (AQ), Wave (Wave 1 vs Wave 2), Emotion (Basic vs. Complex), Experience with Others Wearing Face Coverings (hereafter 'Face Covering': Rarely vs. Sometimes vs. Often) and all the possible interactions between them as fixed effects using the *aov()* function. In our case, we obtained similar findings to that found in the mixed effects model, which are described below.

We found a main effect of AQ ($F(1, 554) = 17.65$, $p < .001$). The correlation between AQ and Emotion Recognition Accuracy was significantly negative, $r(564) = -0.17$, $p < .001$. There was also a main effect of Emotion ($F(1, 554) = 236.98$, $p < .001$), which was qualified by an Emotion × Wave interaction ($F(1, 554) = 9.02$, $p < .003$). Post-hoc comparisons revealed that the difference in performance across Waves was not significant for Basic emotions ($t(944) = 0.58$, $p = .564$) but performance was better for Wave 2 than Wave 1 for Complex emotions ($t(944) = 2.48$, $p = .013$).

Importantly, we also found a significant Face Covering × AQ × Wave interaction ($F(2, 554) = 4.60$, $p = .011$). Examining the effect of AQ at each level of Face Covering by Wave revealed a significant negative AQ effect for Rarely Wave 2 ($t(554) = 2.96$, $p = .003$) and Often Wave 1 ($t(554) = 4.07$, $p < .001$). Direct comparison of the estimated AQ slope between Waves for each Face Covering level revealed a significant difference for the Often condition ($t(554) = 2.33$, $p = .020$), with the slope being more negative for Wave 1 than for Wave 2, and a marginally significant difference for the Rarely condition ($t(554) = 1.97$, $p = .050$), with the slope being more negative for Wave 2 than for Wave 1.

Section S2: Analysis on Wave 1 Data

As stated in the study preregistration protocol, we analysed data from Wave 1 to replicate expected findings (i.e., the negative relationship between autistic traits and emotion recognition accuracy) using both binomial mixed effects model and ANOVA. For the binomial mixed effects model, we fitted a similar model as the main analysis minus the Wave predictor and all its interactions. The output is shown below in Table S2.1.

Table S2.1

Mixed effects model results on Wave 1 data

	χ^2	df	*p*
Intercept	57.39	1	< .001
Emotion	0.47	1	.491
Face Covering	3.46	2	.177
AQ	8.89	1	.003
Emotion × Face Covering	0.14	2	.932
Emotion × AQ	0.00	1	.967
Face Covering × AQ	7.89	2	.019
Emotion × Face Covering × AQ	1.11	2	.575

There was a negative effect of AQ ($\chi^2(1) = 8.89$, $p = .003$; $B = -0.01$, SE = 0.00) as expected, and a Face Covering × AQ interaction ($\chi^2(2) = 7.89$, $p = .019$). Follow up tests revealed that the AQ effect was only significantly negative for the Often condition ($z = 3.94$, $p < .001$), and direct comparisons of the AQ effect between Face Covering levels showed a significant difference only between Often and Rarely ($z = 2.73$, $p = .018$), with the AQ slope being more negative for the former (see Figure S2.1).

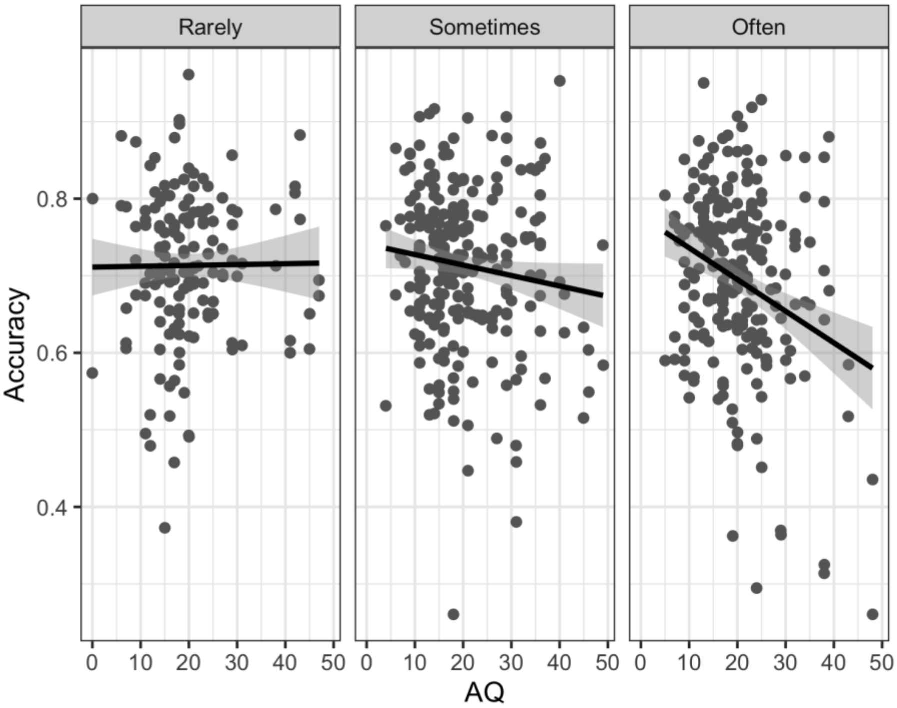

Figure S2.1. Accuracy on the Emotion Recognition Task as a function of autistic traits (AQ) by Experience with Others Wearing Face Coverings (Rarely vs. Sometimes vs. Often)

Results from the ANOVA on Wave 1 data largely corresponded to that found for the mixed effects model: there was a main effect of AQ ($F(1, 302) = 11.24$, $p < .001$) and an interaction between Face Covering and AQ ($F(2, 302) = 4.44$, $p = .013$). Subsequent tests similarly revealed that the negative AQ effect was only significant for the Often condition ($t(302) = 4.18$, $p < .001$), and direct comparisons of the AQ effect between Face Covering levels showed a significant difference only between Often and Rarely ($t(302) = 2.88$, $p = .012$), with the AQ slope being more negative for the former. Additionally, there was also a main effect of Emotion ($F(1, 302) = 95.25$, $p < .001$), with better performance on the Complex emotions than Basic emotions ($t(302) = 9.56$, p < .001) presumably due to fewer labels to choose from for the Complex emotion trials than the Basic emotion trials.

Section S3: Data Analysis using Autism Diagnosis

To examine whether our findings would be replicated using autism diagnosis rather than autistic traits, we ran a similar mixed effects model as reported in the manuscript, with the exception that we replaced AQ (a continuous predictor) with participants' self-reported autism diagnosis (Diagnosis; a categorical predictor). In both waves, approximately 10% of the participants identified as autistic (Wave 1 n = 29; Wave 2 n = 27) whereas the rest were classified as neurotypical (Wave 1 n = 279; Wave 2 n = 231). The results of the mixed effects model can be seen below in Table S3.1.

Table S3.1

Mixed effects model results using autism diagnosis (Diagnosis) instead of autistic traits (AQ).

	χ^2	df	p
Intercept	49.71	1	< .001
Emotion	1.41	1	.235
Face Covering	2.21	2	.331
Diagnosis	7.06	1	.008
Wave	1.47	1	.226
Emotion × Face Covering	0.10	2	.952
Emotion × Diagnosis	3.81	1	.051
Face Covering × Diagnosis	0.67	2	.714
Emotion × Wave	1.34	1	.248
Face Covering × Wave	9.77	2	.008
Diagnosis × Wave	0.72	1	.395
Emotion × Face Covering × Diagnosis	2.29	2	.318
Emotion × Face Covering × Wave	0.93	2	.630
Emotion × Diagnosis × Wave	0.19	1	.664
Face Covering × Diagnosis × Wave	8.65	2	.013
Emotion × Face Covering × Diagnosis × Wave	0.16	2	.924

Generally, the same findings were observed. The Diagnosis predictor ($\chi^2(1) = 7.06$, $p = .008$) and the interaction between Face Covering × Wave ($\chi^2(2) = 9.77$, $p = .008$) were significant, both of which were qualified by a Face Covering × Diagnosis × Wave interaction

($\chi^2(2) = 8.65$, $p = .013$) akin to the three-way interaction of Face Covering × AQ × Wave seen in the main analysis. Pairwise comparisons revealed that improvement across Waves was only observed among autistic participants in the Often condition ($z = 3.41$, $p = .001$; see Figure S3.1).

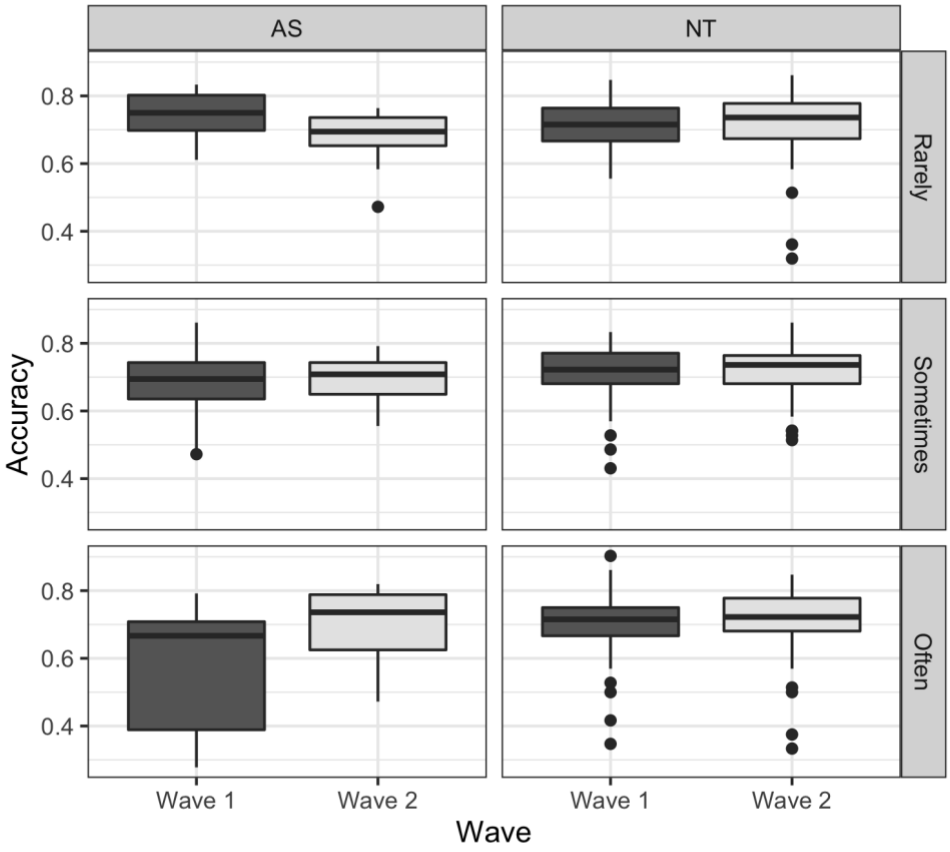

Figure S3.1. Accuracy on the Emotion Recognition Task by Autism Diagnosis (Autistic [AS] vs. Neurotypical [NT]), Wave (Wave 1 vs. Wave 2), and Experience with Others Wearing Face Coverings (Rarely vs. Sometimes vs. Often)

Section S4: Analysis on Wave 2 Data using Autistic Traits and Alexithymia Scores

To examine the relative contribution of autistic traits and alexithymia on emotion recognition ability, we fitted a binomial mixed effects model on Wave 2 data (during which we collected both autistic traits and alexithymia scores) using the following as fixed effects: Emotion (Basic vs. Complex), Experience with Others Wearing Face Coverings (Face Covering; Rarely vs. Sometimes vs. Often), autistic traits (AQ), alexithymia scores (TAS), and all the possible interactions between them. As random effects, we entered random intercept for participant and item, and random by-participant slope for Emotion. The model results are displayed in Table S4.1.

Table S4.1

Mixed effects model results on Wave 2 Data using autistic traits (AQ) and alexithymia scores (TAS).

	χ^2	df	*p*
Intercept	61.65	1	< .001
Emotion	1.61	1	.205
Face Covering	0.23	2	.891
AQ	0.05	1	.819
TAS	7.26	1	.007
Emotion × Face Covering	5.44	2	.066
Emotion × AQ	0.15	1	.702
Face Covering × AQ	0.66	2	.718
Emotion × TAS	4.15	1	.042
Face Covering × TAS	4.14	2	.126
AQ × TAS	0.51	1	.477
Emotion × Face Covering × AQ	6.16	2	.046
Emotion × Face Covering × TAS	9.10	2	.011
Emotion × AQ × TAS	0.12	1	.730
Face Covering × AQ × TAS	1.24	2	.537
Emotion × Face Covering × AQ × TAS	3.85	2	.146

We found that TAS was a significant predictor ($\chi^2(1) = 7.26$, $p = .007$) and the interaction between Emotion and TAS was also significant ($\chi^2(1) = 4.15$, $p = .042$). These two were qualified by a three-way interaction involving Emotion × Face Covering × TAS ($\chi^2(2) = 9.10$, $p = .011$). Subsequent tests revealed that the effect of TAS was significantly negative for Complex emotions in the Rarely condition ($z = 3.38$, $p = .001$) and Basic emotions in the Often condition ($z = 2.41$, $p = .016$). Direct comparisons on the effect of TAS between the emotions for each Face Covering level revealed significant differences in the Rarely ($z = 2.41$, $p = .016$) and Sometimes ($z = 2.11$, $p = .035$) conditions, with the TAS effect more negative in the Complex emotions than in the Basic emotions in both cases (see Figure S4.1).

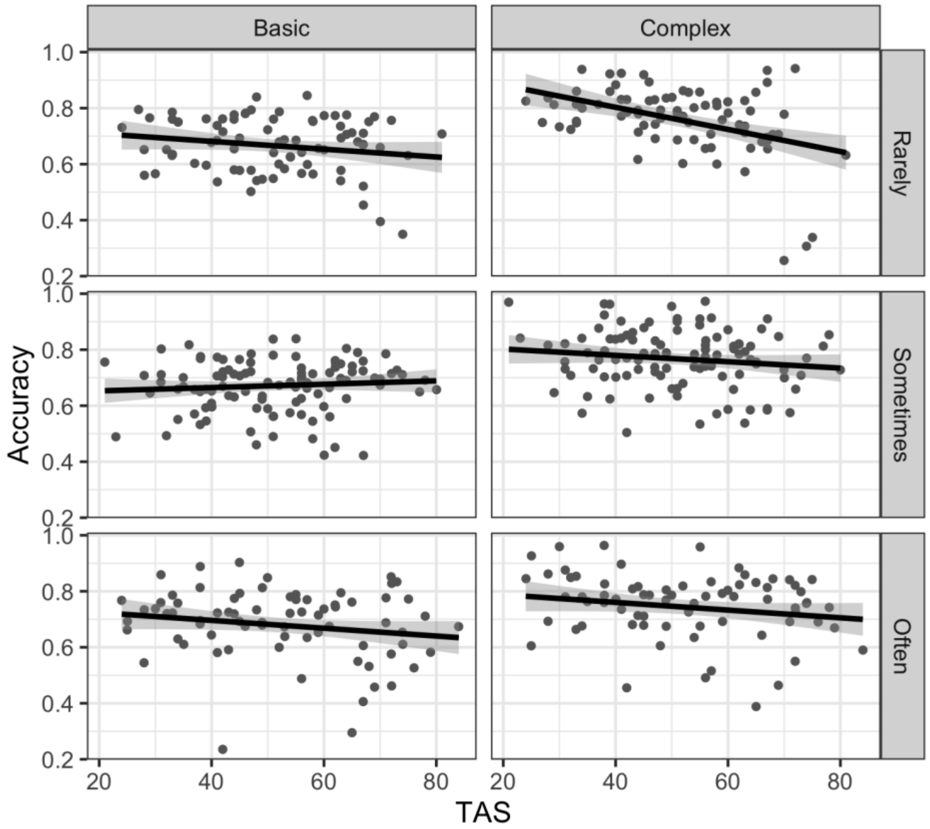

Figure S4.1. Accuracy on the Emotion Recognition Task as a function of alexithymia scores (TAS) by Emotion (Basic vs. Complex) and Experience with Others Wearing Face Coverings (Rarely vs. Sometimes vs. Often)

Additionally, there was also a three-way Emotion × Face Covering × AQ interaction ($\chi^2(2) = 6.16$, $p = .046$). Subsequent tests for the effect of AQ by each level of Emotion and Face Covering revealed no significant effect of AQ, but the slope for AQ was significantly more negative for the Complex emotions relative to the Basic emotions only in the Often condition ($z = 2.31$, $p = .021$; see Figure S4.2).

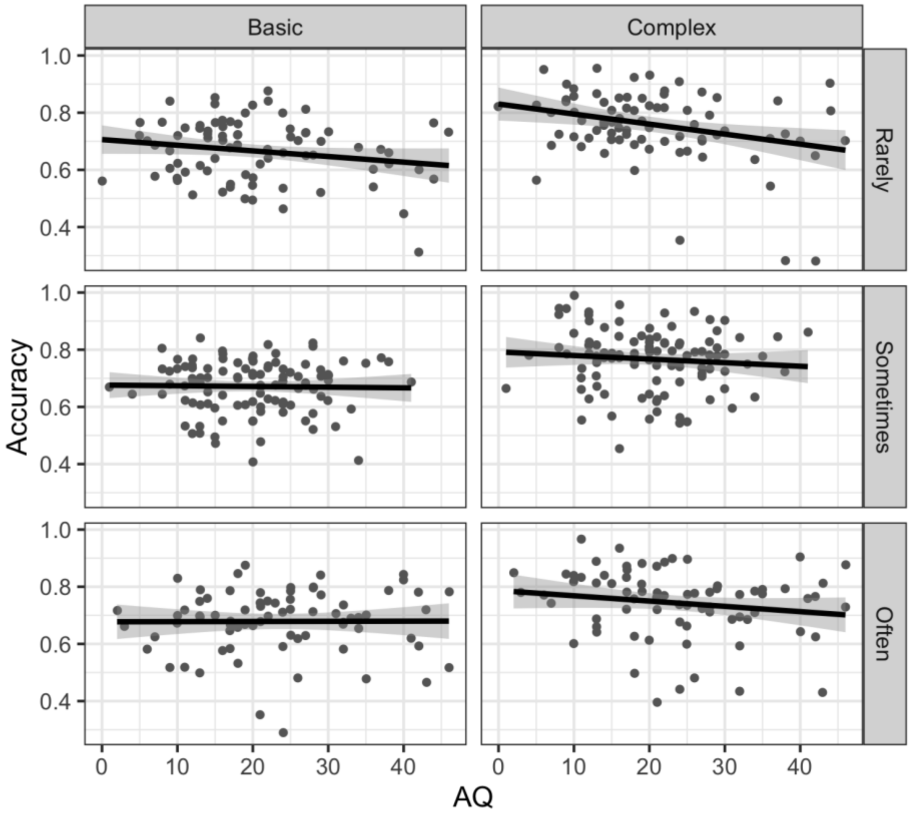

Figure S4.2. Accuracy on the Emotion Recognition Task as a function of autistic traits (AQ) by Emotion (Basic vs. Complex) and Experience with Others Wearing Face Coverings (Rarely vs. Sometimes vs. Often)

References

Jaeger, T. F. (2008). Categorical data analysis: Away from ANOVAs (transformation or not) and towards logit mixed models. *Journal of Memory and Language*, *59*(4), 434–446. https://doi.org/10.1016/j.jml.2007.11.007.Categorical
